# Identification of Novel Small RNAs and Characterization of the 6S RNA of *Coxiella burnetii*

**DOI:** 10.1371/journal.pone.0100147

**Published:** 2014-06-20

**Authors:** Indu Warrier, Linda D. Hicks, James M. Battisti, Rahul Raghavan, Michael F. Minnick

**Affiliations:** 1 Division of Biological Sciences, University of Montana, Missoula, Montana, United States of America; 2 Department of Biology, Portland State University, Portland, Oregon, United States of America; Texas A& M Health Science Center, United States of America

## Abstract

*Coxiella burnetii*, an obligate intracellular bacterial pathogen that causes Q fever, undergoes a biphasic developmental cycle that alternates between a metabolically-active large cell variant (LCV) and a dormant small cell variant (SCV). As such, the bacterium undoubtedly employs complex modes of regulating its lifecycle, metabolism and pathogenesis. Small RNAs (sRNAs) have been shown to play important regulatory roles in controlling metabolism and virulence in several pathogenic bacteria. We hypothesize that sRNAs are involved in regulating growth and development of *C. burnetii* and its infection of host cells. To address the hypothesis and identify potential sRNAs, we subjected total RNA isolated from *Coxiella* cultured axenically and in Vero host cells to deep-sequencing. Using this approach, we identified fifteen novel *C. burnetii* sRNAs (CbSRs). Fourteen CbSRs were validated by Northern blotting. Most CbSRs showed differential expression, with increased levels in LCVs. Eight CbSRs were upregulated (≥2-fold) during intracellular growth as compared to growth in axenic medium. Along with the fifteen sRNAs, we also identified three sRNAs that have been previously described from other bacteria, including RNase P RNA, tmRNA and 6S RNA. The 6S regulatory sRNA of *C. burnetii* was found to accumulate over log phase-growth with a maximum level attained in the SCV stage. The 6S RNA-encoding gene (*ssrS*) was mapped to the 5′ UTR of *ygfA*; a highly conserved linkage in eubacteria. The predicted secondary structure of the 6S RNA possesses three highly conserved domains found in 6S RNAs of other eubacteria. We also demonstrate that *Coxiella’s* 6S RNA interacts with RNA polymerase (RNAP) in a specific manner. Finally, transcript levels of 6S RNA were found to be at much higher levels when *Coxiella* was grown in host cells relative to axenic culture, indicating a potential role in regulating the bacterium’s intracellular stress response by interacting with RNAP during transcription.

## Introduction

During infection, pathogenic bacteria must adapt to diverse and dynamic environments imposed by their host and regulate synthesis of a variety of molecules (DNA, RNA and proteins) needed to colonize, replicate and persist. This kind of regulation must be rapid, metabolically inexpensive and efficient. There is growing evidence that post-transcriptional control mediated by small RNAs (sRNAs) plays a significant role in bacterial regulation [1], [2]. In pathogenic bacteria, sRNAs are known to coordinate virulence gene expression and also stress responses that are important for survival in the host [3], [4]. Bacterial sRNAs are typically 100–400 bases in length and are categorized as *cis*-encoded sRNAs and *trans*-encoded sRNAs. Most *cis*-encoded sRNAs are located within 5′ untranslated regions (UTRs) of mRNAs and are transcribed in the antisense orientation to the corresponding mRNA. *Cis*-encoded sRNAs can expose or block a ribosome-binding site (RBS) by adopting different conformations in response to various environmental cues, thereby regulating translation. On the other hand, *trans*-encoded sRNAs are located in intergenic regions (IGRs). They share only limited complementarity with their target RNAs and are thought to regulate translation and/or stability of these RNAs [2]. sRNAs can interact with mRNA or protein in order to bring about regulation, but a majority of them function by binding to mRNA targets. An example of a widely distributed and well-studied sRNA is 6S RNA. 6S RNA binds to RNA polymerase (RNAP)-σ^70^ complex and regulates transcription by altering RNAP’s promoter specificity during stationary phase [5], [6].

*Coxiella burnetii*, the causative agent of Q fever, is classified as a Gram-negative obligate intracellular γ-proteobacterium. Human Q fever is generally a zoonosis acquired by inhalation of contaminated aerosols and can present either as an acute or chronic disease. An acute case of Q fever typically ranges from an asymptomatic infection to an influenza-like illness accompanied by high fever, malaise, atypical pneumonia, myalgia and hepatitis. In approximately 2–5% of cases, chronic Q fever occurs and manifests as endocarditis, especially in patients with predisposing valvular defects [7]. The pathogen’s biphasic developmental cycle consists of two cellular forms. An infectious, dormant small cell variant (SCV) is spore-like and can endure adverse environmental conditions such as heat, pressure, UV light and desiccation. Following inhalation, *Coxiella* enters alveolar macrophages by endocytosis and generates a phagolysosome-like vacuole termed a parasitophorus vacuole (PV). The PV interacts with autophagosomes for bacterial nutrition [8]. At approximately 8 h post-infection, SCVs metamorphose to form metabolically active LCVs in the PV, with a doubling time of approximately 11 hours [9], [10]. Following 6–8 days of intracellular growth, the PV reaches maturity and occupies almost the entire volume of the cell, and it is filled with a mixture of LCVs and SCVs. By approximately 12 days, the entire bacterial population has transformed into SCVs that are eventually released upon lysis of the host cell [10].

*C. burnetii* encounters various and sudden changes in environmental conditions during its life cycle, including a rapid upshift in temperature upon transmission from contaminated aerosols to the human lung, and a downshift in pH and an increase in reactive oxygen intermediates (ROIs) in the PV. All of these events are relevant to rapid, sRNA-mediated regulation [2]. Recent reports have identified sRNAs in a variety of pathogenic bacteria, including *Legionella pneumophila* [11] and *Streptococcus pyogenes* [12]. Reports have also shown the involvement of sRNAs in the pathogenesis of *Streptococcus pneumoniae*, *Salmonella spp., Yersinia pseudotuberculosis* and *Listeria monocytogenes* [13]–[16].

The sRNAs of intracellular bacterial pathogens are poorly characterized, and there are no reports on sRNAs of *C. burnetii*. Thus, the aim of our study was to identify sRNAs associated with the bacterium’s developmental cycle and host cell infection. Here, we describe a set of 15 novel *Coxiella* sRNAs identified by high-throughput sequencing of RNA (RNA-seq) isolated from distinct life stages and culture conditions. We also characterized the 6S sRNA of *C. burnetii* in an effort to elucidate the function of one of the sRNA’s identified. We found that 6S RNA specifically binds to *Coxiella’s* RNA polymerase (RNAP), reaches its highest concentration in SCVs, and its expression is markedly increased during intracellular versus axenic growth.

## Materials and Methods

### Cultivation of *C. burnetii*

*C. burnetii* Nine Mile phase II (strain RSA439, clone 4) was propagated in African green monkey kidney (Vero) fibroblast cells (CL-81; American Type Culture Collection) grown in RPMI medium (Invitrogen Corp.) supplemented with 10% fetal bovine serum at 37°C in a 5% CO_2_ atmosphere. Bacteria were purified from host cells using differential centrifugation, as previously described [17]. LCVs were harvested at 72 h post-infection from infected cells using digitonin [18]. SCVs were harvested and prepared at 21 days post-infection (dpi), as previously described [19], and used to infect Vero cell monolayers for the production of synchronized bacterial cultures. *C. burnetii* was also cultivated axenically in ACCM2 at 37°C in a tri-gas incubator (2.5% O_2_, 5% CO_2_, 92.5% N_2_) with continuous shaking at 75 RPM [20]. To generate LCVs, ACCM2 was inoculated with 10-d-old ACCM2-cultured bacteria, incubated 72 h, and isolated by centrifugation (10,000×g for 20 min at 4°C), as previously described [9]. SCV generation in ACCM2 was identical to LCVs except bacteria were grown for 7 d, and then flask lids were tightened and cultured an addition at 14 d on the lab bench (∼25°C) without replenishing the medium [21].

### RNA Isolation and Deep-sequencing

To isolate *C. burnetii* RNA from infected Vero cells, LCVs were prepared as above and treated with 40 µg/ml RNase A in RNase A digestion buffer [10 mM Tris-Cl (pH 7.5), 50 mM NaCl, 5 mM EDTA] to reduce host cell RNA contamination. SCVs were prepared as above and used directly. Total RNA used in deep-sequencing was purified from LCVs and SCVs with a Ribopure kit (Ambion). Resulting RNAs were treated with excess DNase I to remove trace amounts of residual DNA using a DNA-*free* kit, as instructed by the manufacturer (Applied Biosystems). RNA was precipitated with 100% ethanol and enriched for bacterial RNAs by sequential use of MICROB*Enrich* (Ambion), MICROB*Express* (Ambion) and Ribo-Zero (Epicentre) kits to increase the relative level of *C. burnetii* RNA derived from Vero cell-propagated organisms and to exclude rRNAs, respectively. RNA from *C. burnetii* cells cultured in ACCM2 was done as for infected Vero cells, however, the MICROB*Enrich* (Ambion) step was omitted. RNA was quantified using a NanoPhotometer (Implen) and checked for integrity using a 2100 Bioanalyzer (Agilent Technologies). Sequencing libraries were prepared with a TruSeq RNA sample preparation kit (Illumina). Libraries were sequenced on an Illumina HiSeq 2000 (76 cycles) at the Yale Center for Genome Analysis (West Haven, CT). Two independent samples were sequenced from all conditions, and sequencing statistics are given in Table S1. Deep sequencing data were submitted to the Sequence Read Archive (SRA) database, NCBI, and assigned the accession number SRP041556.

### Mapping of Sequencing Reads and Quantification of Transcripts

Sequencing reads were mapped on the *C. burnetii* Nine Mile Phase I (RSA 493) genome (NC_002971.3) using BWA software [22]. The algorithm was set to allow for two mismatches between 76-nt reads and the genome sequence. Coverage at each nucleotide position was visualized using Artemis software [23]. Expression values for each genomic location were calculated by determining the number of reads overlapping that region and normalizing it to the total number of reads in each library and the region’s length. The average expression values obtained from two independent libraries per time point were denoted as Mean Expression Values (MEVs). Transcripts were qualified as sRNAs if they were 50–400 nt in length, had an MEV≥5 times that of the flanking 50 nucleotides and did not correspond exactly to an annotated open reading frame (ORF). The presence of σ^70^ consensus promoters and rho-independent terminators was predicted using BPROM [24] and TranstermHP [25] software, respectively.

### Northern Blot Analysis

Northern blots were carried out using a NorthernMax kit (Ambion) as per manufacturer’s instructions. Briefly, total RNAs of *C. burnetii* grown in Vero cells or ACCM2 were isolated by sequential use of Ribopure (Ambion) and DNA-*free* (Applied Biosystems) kits and then precipitated with 100% ethanol. For quality control purposes, RNA samples were occasionally analyzed on denaturing acrylamide gels to check for RNA integrity. RNA degradation was not observed in samples used in the study (data not shown). RNA (3 µg per lane, except CbSR 2, where 1.7 µg RNA was used) was electrophoresed through 1.5% agarose-formaldehyde gels and blotted onto positively-charged BrightStar-Plus nylon membranes (Ambion). Membranes were then UV-cross-linked or chemically cross-linked by 1-ethyl-3-(3-dimethylaminopropyl) carbodiimide (EDC) (Sigma-Aldrich), as previously described [26]. Hybridizations were carried out using single-stranded RNA probes specific to each sRNA. RNA probes were generated by T7 promoter-mediated *in vitro* transcription of PCR products using a MEGAscript kit as instructed (Ambion), in the presence of biotin-labeled UTP (Bio-16-UTP; Ambion). Finally, membranes were developed with a BrightStart BioDetect kit (Ambion) following the manufacturer’s protocol, and visualized using a LAS-3000 imaging system (Fujifilm). Densitometry was performed using ImageJ software [27]. [Please see Table S2 for probe details].

### Quantitative PCR (qPCR) and Quantitative Real-Time PCR (qRT-PCR)

qPCR was performed as previously described [19] using a primer set specific to *C. burnetii’s rpoS* gene for generation of a growth curve showing genome numbers as a function of time [9]. Primers specific to *C. burnetii’s* 6S RNA encoding gene (*ssrS*) were designed using Beacon Designer 7.5 software (Biosoft International). qRT-PCR data were obtained with a 6S RNA primer set and normalized to corresponding *C. burnetii* genome numbers. [Please see Table S3 for primer details].

### *C. burnetii* Extract Preparation

A mixed population (11 dpi) of *C. burnetii* grown in Vero cells was pelleted by centrifugation (10,000×g for 10 min at 4°C) and resuspended in 250 µl Net2 buffer [50 mM Tris (pH 7.4), 150 mM NaCl, 0.05% NP-40 (triton X-100)] supplemented with protease inhibitor (Complete Mini Protease inhibitor cocktail tablets used as instructed; Roche). RNasin Plus (Promega) was added to a final concentration of 1 U/µl and bacteria were lysed by five alternating freeze-thaws cycles in liquid nitrogen and a 37°C water bath (5 min each). The resulting lysate was clarified by centrifugation (10,000×g for 10 min at 4°C), and the supernatant was used for further analysis.

### Immunoprecipitation (IP)

Protein A Sepharose (PAS) beads (CL-4B; GE Healthcare) were swelled (2 mg PAS in 100 µl Net2 buffer) for 30 min at room temperature and washed three times with 400 µl cold Net2 buffer followed by centrifugation (400×g for 30 sec). IPs were carried out using rabbit anti-*Escherichia coli* RNAP core polyclonal antibody (a generous gift from Dr. Karen Wassarman, University of Wisconsin-Madison), a corresponding rabbit pre-immune serum or rabbit anti-*Coxiella* Com1 polyclonal antibody. Antibodies were incubated with 100 µl PAS-Net2 at a 1∶50 dilution for 16 h at 4°C with gentle agitation. PAS-antibody conjugates were then washed five times with 400 µl cold Net2 buffer as above. *C. burnetii* extract (25 µl) was added to each PAS-antibody conjugate and incubated for 2 h at 4°C with rocking. IP reactions were separated by centrifugation, and PAS beads and supernatants were retained for further analysis. PAS beads were washed five times as above, and the final pellet resuspended in Net2 buffer (200 µl). Approximately 20% of this IP suspension was used for protein analysis and 80% for RNA analysis.

### Protein Analysis

IP beads and supernatants were mixed with equal volumes of 2X Laemmli sample buffer, boiled for 5 min and centrifuged 1 min at 16,000×g. The resulting supernatants were resolved on a 10% acrylamide SDS-PAGE gel. The gel was immediately blotted onto a nitrocellulose membrane (0.45 µm pore size) and blocked for 2 h at room temperature in blocking buffer [5% nonfat dry milk in TBS-T (25 mM Tris-HCl, pH 8.0; 125 mM NaCl; 0.1% Tween 20)] with rocking. Blots were subsequently probed for 16 h with a 1∶2000 dilution of anti-RNAP antibody in antibody binding buffer (TBS-T containing 1% nonfat dry milk) followed by 5 washes of 10 min each in TBS-T. Blots were then incubated for 1 h at room temperature with rocking in a 1∶2000 dilution of peroxide-conjugated goat anti-rabbit IgG antibodies (Sigma) in antibody binding buffer, followed by 5 washes (10 min each) in TBS-T. Finally, blots were developed using a chemiluminescent substrate as instructed by the manufacturer (SuperSignal West Pico kit, Thermo Scientific) and visualized using a LAS-3000 imaging system (Fujifilm).

### RNA Extraction and RNase Protection Assay (RPA)

Total RNA from IP beads and supernatant was isolated by extraction with phenol:chloroform:isoamyl alcohol [25∶24∶1; v/v; (pH 8–8.3)] (Invitrogen) followed by ethanol precipitation. Purified RNA was processed using an RNase Protection assay (RPA) III kit (Ambion) as per manufacturer’s instructions. Specifically, 43 ng of RNA and 4.3 pg of probe were used in each reaction, except in the IP from the anti-Com1 antibody, where 22.8 ng RNA was used. The 6S RNA probe prepared for Northern blot analysis was also used in RPAs. RPA reactions were resolved on gels (5% acrylamide; 8 M urea), transferred to BrightStar-Plus nylon membrane (Ambion) and UV-cross-linked. RPA blots were developed using a BrightStar BioDetect kit as instructed (Ambion) and visualized with a LAS-3000 imaging system (Fujifilm) [Please see Table S4 for probe details].

## Results

### Identification of *C. burnetii* sRNAs

To investigate the transcriptome profile of *C. burnetii* and to identify potential sRNAs, we first isolated RNA from LCVs and SCVs co-cultured in Vero cells as well as those cultured axenically in ACCM2 medium. cDNAs prepared from these RNAs were subjected to Illumina sequencing. This deep sequencing analysis resulted in roughly 23 to 32 million reads from RNA isolated from *C. burnetii* cultured axenically, and ∼47 to 81 million reads from total RNA isolated from Vero co-cultures. On the whole, sequencing reads obtained from *C. burnetii* cultured in ACCM2 mapped well to the genome (97%). On the other hand, sequencing reads from total RNA isolated from bacteria cultured in Vero host cells mapped 76% and ∼72.5% to the genome, respectively (Table S1). By analyzing the sequencing reads, we identified a total of 15 novel sRNAs, which will hereafter be referred to as CbSRs (*Coxiella burnetii* small RNAs) (Table 1).

**Table 1:** Novel *C. burnetii* sRNAs (CbSRs) identified by RNA-seq.

sRNA	Left End^a^	Right End^a^	Size (nt)	Strand	Axenic LCV	Axenic SCV	Vero LCV	Vero SCV
					Mean Expression Value (MEV)			
CbSR 1	12005	12117	113	F	15444.89	257.96	59710.85	711.00
CbSR 2	75261	75503	243	R	7381.43	782.49	15719.76	5885.67
CbSR 3	481609	481806	198	R	577.92	757.68	1703.55	1225.17
CbSR 4	544387	544582	196	R	1392.97	644.14	4163.36	3433.46
CbSR 5	702095	702304	210	R	3302.63	293.32	584.04	338.12
CbSR 6	727878	728097	220	R	1776.92	332.35	778.38	401.85
CbSR 7	657100	657198	99	F	808.27	158.84	103.52	176.22
CbSR 8	866381	866666	286	R	8384.18	338.06	1867.89	508.76
CbSR 9	973800	974012	213	F	370.36	276.08	763.43	484.25
CbSR 10	1090006	1090228	223	R	7138.08	914.10	5713.77	1768.32
CbSR 11	1327797	1328052	256	F	6428.52	922.77	4258.87	4309.17
CbSR 12	1403153	1403300	148	R	4423.01	1477.31	434177.60	35288.85
CbSR 13	1816997	1817305	309	F	3716.12	1186.83	1621.83	1206.80
CbSR 14	1838698	1838886	189	R	790.92	451.29	3719.02	691.73
CbSR 15	1878295	1878543	249	F	1054.67	298.45	205.01	396.01

a Numbering according to NC_002971.3.

**Figure 1:**
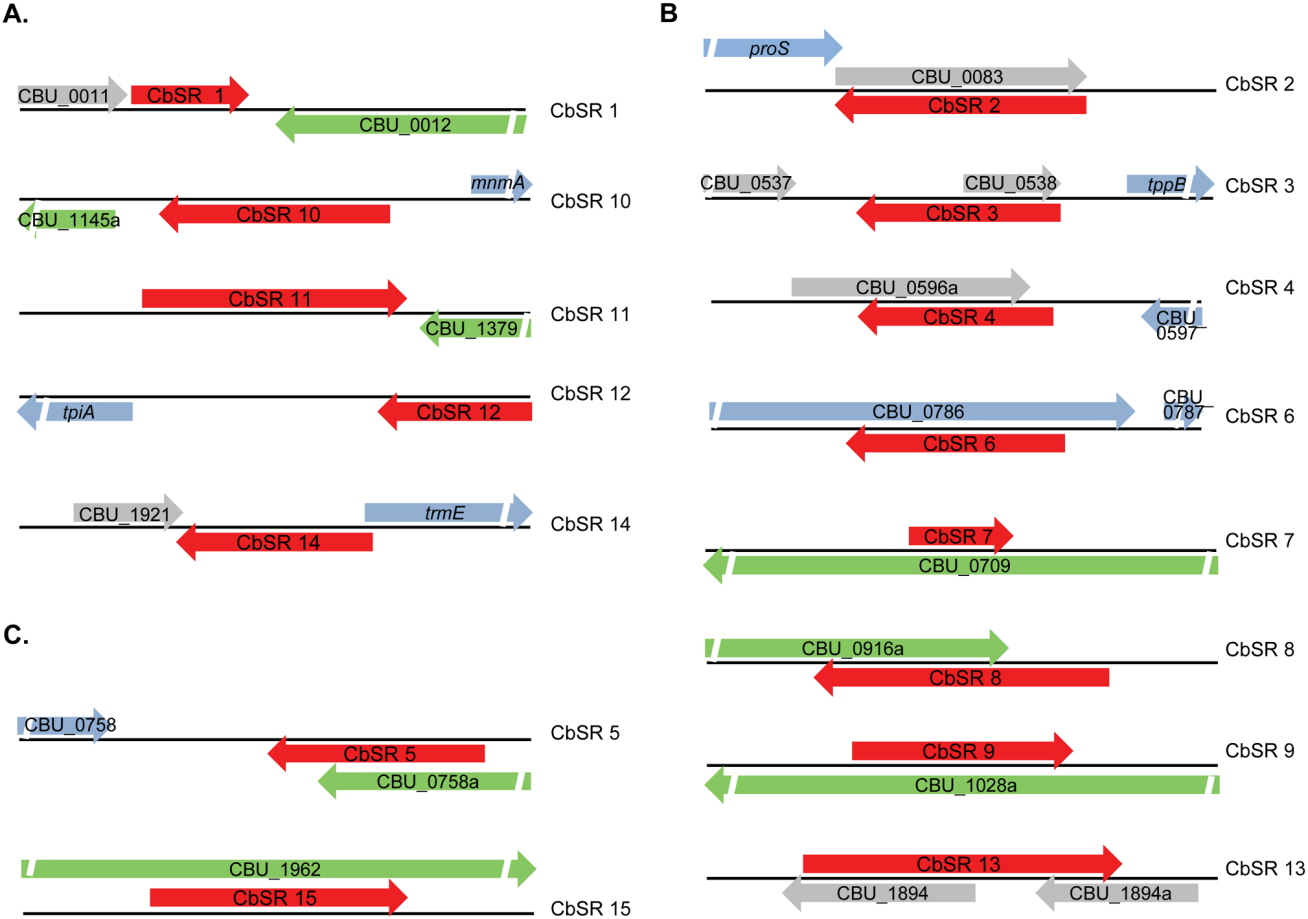
Linkage maps showing CbSR loci on the *C. burnetii* chromosome (black line). Red arrows indicate CbSRs and their relative orientation. Blue, grey and green arrows represent annotated, hypothetical ORFs and pseudogenes, respectively. CbSRs are classified into three groups based on their location relative to adjacent genes: **A.** Group I: CbSRs encoded within IGRs, **B.** Group II: CbSRs located antisense to identified ORFs and **C.** Group III: CbSRs that are ORF-derived.

All 15 CbSRs were present in both LCVs and SCVs cultured in axenic medium as well as in Vero cells. Comparison of the MEVs of LCVs and SCVs indicates that most CbSRs are present at higher levels in LCVs regardless of culture conditions. CbSRs could be classified into three groups based on the relative location of their coding sequence on the genome. Specifically, group I includes sRNAs encoded entirely within an IGR; group II consists of sRNAs situated antisense to identified ORFs (antisense sRNA), and group III includes sRNAs that are ORF-derived (Fig. 1). A majority (eight of fifteen) of the identified sRNAs were antisense sRNAs. Sizes of the CbSRs ranged from 99–309 nt with a minimum MEV of ∼104 and a maximum MEV of ∼434,178 in at least one growth condition. BLAST analyses showed that all sRNAs were found only in *Coxiella* and most sRNAs were highly conserved within six available *C. burnetii* genomes (RSA 493, RSA 331, Dugway 5J108-111, Cb175 Guyana, CbuK Q154 and CbuG Q212) with ≥97% sequence identity. The exception was CbSR 8, which was only found in *C. burnetii* strains RSA493, RSA331 and Cb175 Guyana. Regions immediately upstream of all sRNAs possessed predicted σ^70^ consensus promoters (Table 2), and intrinsic (Rho-independent) terminators were predicted just downstream of seven sRNAs (Table 3), suggesting that these are bona fide sRNAs.

**Table 2:** Putative σ^70^ promoters of CbSRs identified upstream of sRNA coding sequences using BPROM [24].

sRNA	−35 box	−10 box	sRNA	−35 box	−10 box
CbSR 1	TTTATA	GATTGT	CbSR 9	TTTAAT	TACACT
CbSR 2	TTTAAA	TATATT	CbSR 10	TTGTCT	TATAAT
CbSR 3	TTCTAA	CAGGAT	CbSR 11	TTTCAA	TATCTT
CbSR 4	TTGAGA	TAGTCT	CbSR 12	TTGTTA	TATATT
CbSR 5	TTATCA	TGAAAT	CbSR 13	TTGGAG	TATAAT
CbSR 6	TGGCCA	TATAAT	CbSR 14	TTGCTA	TAAAAA
CbSR 7	TTCACA	GATAAT	CbSR 15	TTATCA	GATAAT
CbSR 8	TGGCCA	TATAAT			

**Table 3:** Rho-independent terminators of CbSRs identified using TranstermHP [25]. Portions of the sRNA sequences that overlap with the predicted terminators are underlined.

sRNA	Predicted terminator sequence
CbSR 1	AGGGATCACCAACCCGGGGT GGTTATAGCAACCACCCCTTTTTTTTATTATTA
CbSR 2	CGCCTCAGTATGAAAGAAATCTCGGCCGTTGATGTCCGAGATTTCTTCAT CTAAACACAG
CbSR 3	AAAGCCTAAGAAAAGCGCCATCGGTGTTTTTCTTAGCCCCC
CbSR 10	ATCTACGTAAACAAAGCAGG CAAAATCCTCGAATCGGATCTGCCTGCTTTTTTTTGAAGAAA
CbSR 11	TGATTATTTCCCCCAGCCTAGTCTGTC CGTTGTAAAACGGCAGCTAGGCTGCTTTCATTCCAGG
CbSR 12	TTGTACTAATAAAGAGGACCGCTTTTGCGGTCCTTTTTTTTCTCACTT
CbSR 13	GAGGGGCTTGAAGAACACTAACGGTGTTTTTCTTAGCTCCT

### Verification of sRNA Candidates

**Figure 2:**
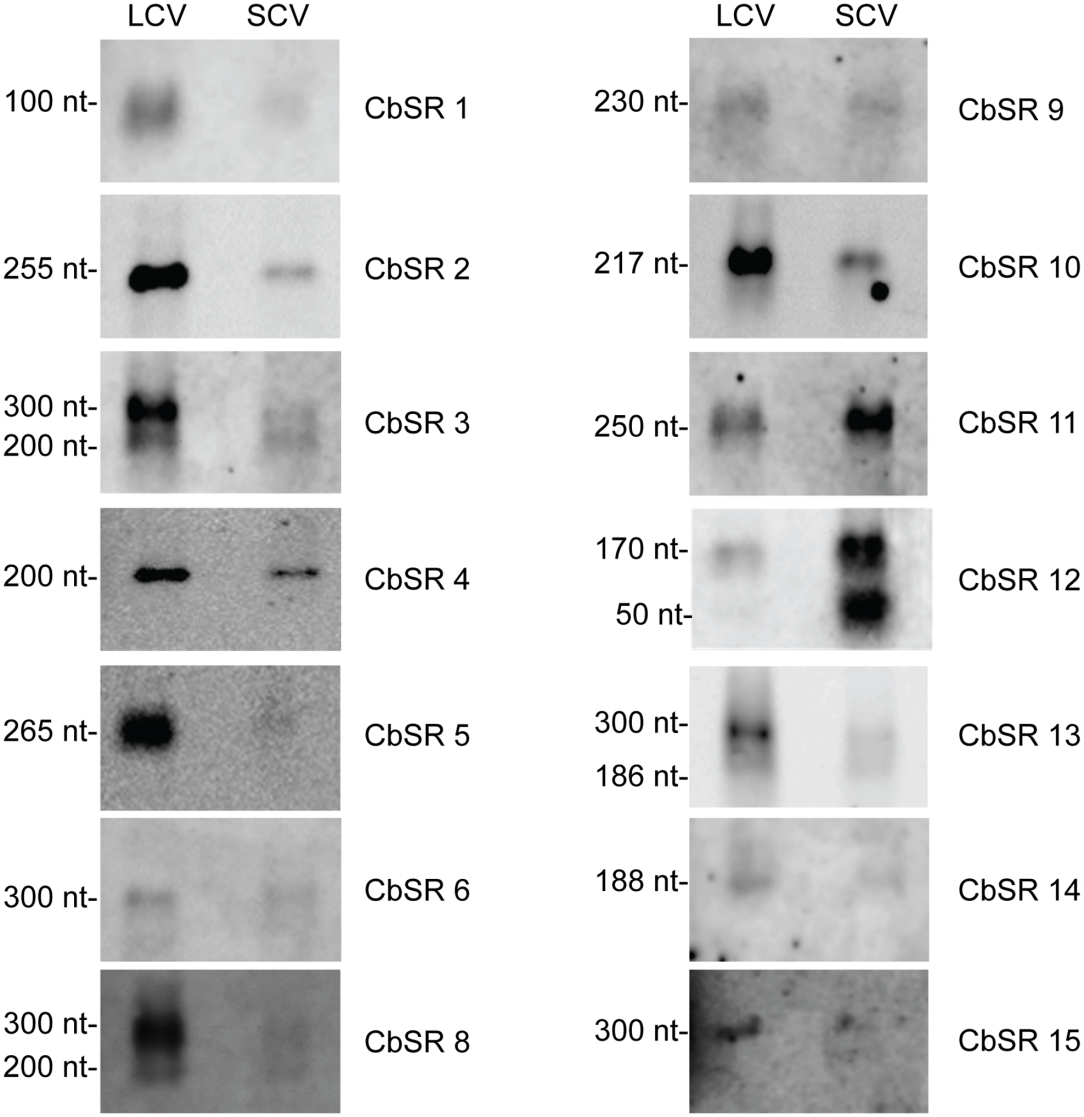
Northern blot detection of CbSRs. RNA was isolated from LCVs (3 dpi) and SCVs (21 dpi) grown in ACCM2. Hybridizations were performed at high stringency using biotinylated oligonucleotide probes specific to each CbSR. 3 µg RNA was used for all lanes. Apparent sizes of the CbSRs, as calculated from Northern blots, are indicated. (Note: intensity of bands is not comparable between panels, since exposure times for each panel have not been optimized).

The fifteen sRNA candidates identified by RNA-seq were further analyzed by Northern blotting of total RNAs from *C. burnetii* LCV or SCV morphotypes (Fig. 2). Northern blots were probed using strand-specific biotinylated RNA oligonucleotides specific to each sRNA. In each case, CbSRs produced distinct bands on the Northern blots, validating their existence in the transcriptome as well as the strand of origin in the *C. burnetii* genome. However, CbSR 7 was not observed. We believe this was due to relatively low CbSR 7 transcript quantity which was undetectable by Northern analysis [28].

For most of the CbSRs, estimated band sizes on Northern blots corresponded to the sRNA lengths predicted by RNA-seq analysis. However, four out of fourteen CbSRs showed multiple bands on blots (e.g., CbSR 3, CbSR 8, CbSR 12 and CbSR 13). First, in the case of CbSR 3, a longer transcript (∼300 nt) was observed, which could represent a primary transcript that is cleaved to give a mature sRNA of ∼200 nt, as obtained by RNA-seq. A similar processing has been previously described for sRNAs of other bacteria [29], [30]. Second, CbSR 8, CbSR 12 and CbSR 13 Northern blots revealed two bands in which the larger bands corresponded to sizes obtained by RNA-seq, suggesting that the upper band is the actual sRNA. In CbSR 12, the molar ratios of the two different-sized bands observed varied between the LCV and SCV stages, possibly indicating different RNA processing, similar to what occurs with the SroF sRNA of *E. coli* [30]. When transcript levels of the fifteen CbSRs were compared on blots, most had increased expression during the metabolically-active LCV phase with exceptions like CbSR 9 which was present in seemingly equal amounts in both morphotypes.

Northern blot signal intensity of most CbSRs corresponded to the MEVs obtained by RNA-seq (Table 1), with a few anomalies like CbSR 11, CbSR 12 and CbSR 13. Although the larger band (∼300 nt) of CbSR 3 doesn’t correspond well with RNA-seq MEV ratios, the lower (∼200 nt) band does. Furthermore, signal intensities of CbSR 11 and CbSR 12 bands on Northern blots, as a function of morphotype, were consistently reversed relative to their deep sequencing MEVs with increased transcript level in SCVs rather than LCVs. Further investigation is required to determine the basis of these discrepancies.

### sRNAs Up-regulated during Intracellular Growth

**Figure 3:**
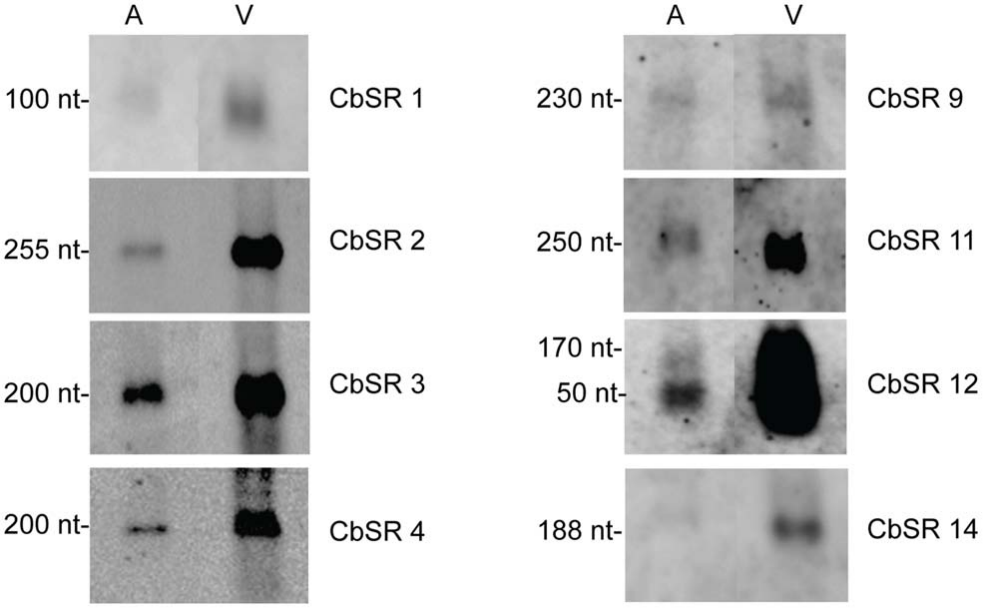
Northern blots showing CbSRs up-regulated (≥2 fold) in host cells relative to ACCM2. RNA was isolated from SCVs (3 dpi) grown in ACCM2 (A) and in Vero host cells (V). Hybridizations were performed at high stringency using biotinylated oligonucleotide probes specific to each CbSR. 3 µg RNA was used for all lanes. Apparent sizes of the CbSRs, as calculated from the Northern blots, are indicated. (Note: intensity of bands is not comparable between panels, since exposure times for each panel have not been optimized).

To search for sRNA regulators that are significantly up-regulated during a host cell infection, we compared expression levels of CbSRs from *C. burnetii* cultured in Vero host cells to those cultured axenically in ACCM2 (Table 1). Results showed eight CbSRs with increased MEVs in host cells (i.e., at least 2-fold higher) relative to axenic medium, including CbSR 1, CbSR 2, CbSR 3, CbSR 4, CbSR 9, CbSR 11, CbSR 12 and CbSR 14. Northern hybridizations were performed on each of these CbSRs to confirm their existence and determine their levels under different growth conditions (Fig. 3). The results observed were consistent with RNA-seq data. CbSR 12 showed a marked 24-fold higher level in Vero-grown *C. burnetii* suggesting a possible role in regulating a bacterial response related to intracellular survival. Other CbSRs that were markedly increased during intracellular growth included CbSR 2 and CbSR 4, which were 8-fold and 5-fold higher by MEV, respectively, compared to values obtained from axenically-grown *C. burnetii*.

### Identification and Characterization of *C. burnetii’s* 6S RNA

**Figure 4:**
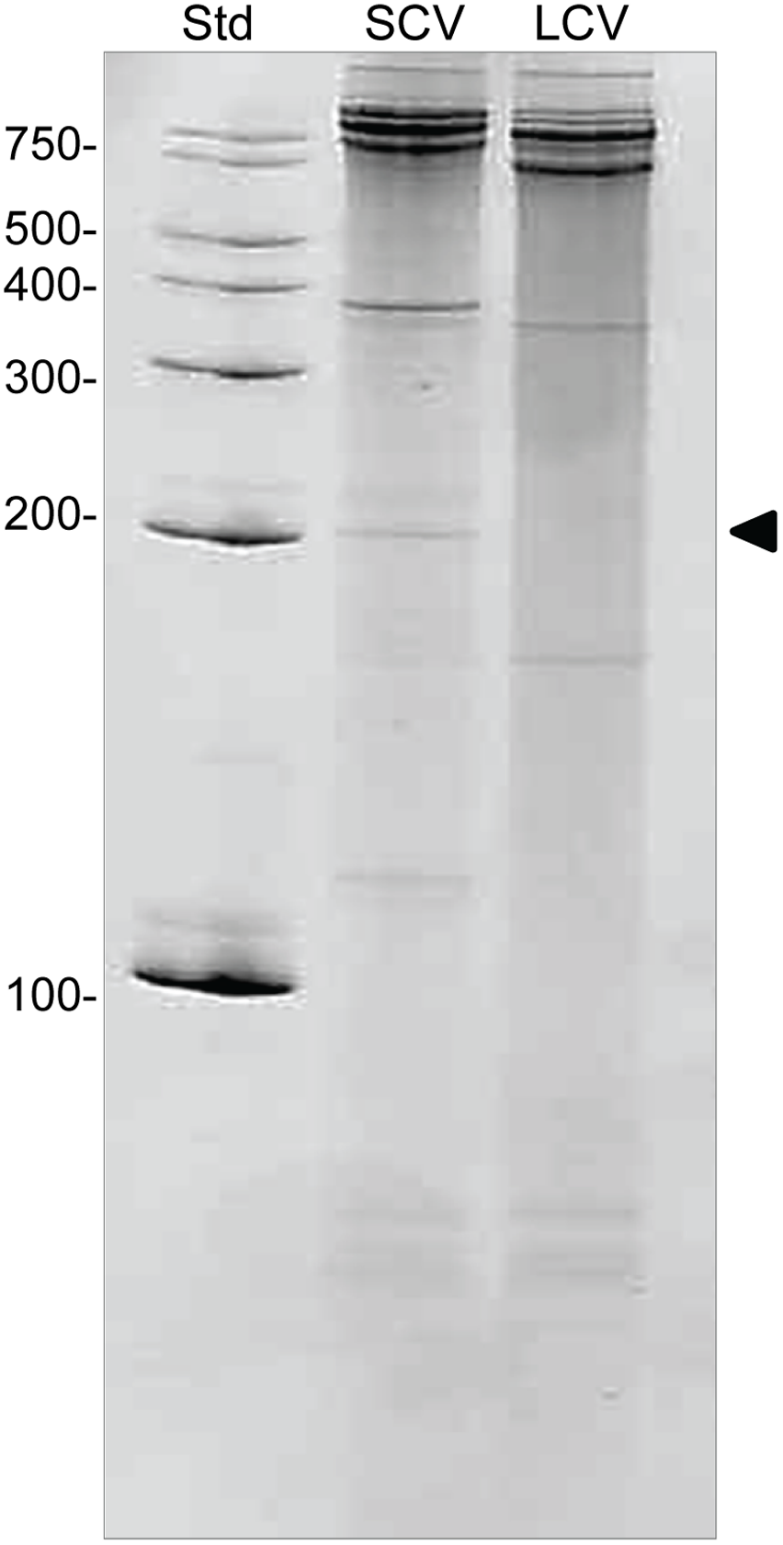
*C. burnetii* total RNA separated on a denaturing gel. RNA isolated from *C. burnetii* LCVs (3 dpi) and SCVs (14 dpi) grown in Vero host cells, separated on a denaturing 8 M urea 8% acrylamide gel stained with ethidium bromide (5 µg RNA per lane). Arrow indicates the position of 6S RNA at ∼200 nucleotides. The number of nucleotides in RNA size standards (Std) is indicated to the left.

**Figure 5:**
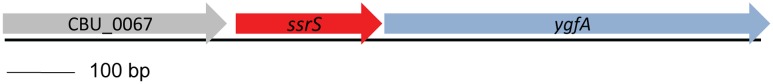
Linkage map showing the location of *C. burnetii’s* 6S RNA gene (*ssrS*). *ssrS* is encoded in the 5′, untranslated region (UTR) of *ygfA* (encoding formyl tetrahydrofolate cyclo-ligase; CBU_0066). The gene immediately upstream (CBU_0067) encodes a hypothetical protein.

When total RNAs from LCV and SCV morphotypes of *C. burnetii* were analyzed on a urea-denaturing acrylamide gel, a prominent band of ∼200 nt was consistently observed in SCV, but not LCV, RNA (Fig. 4). Since previous studies have shown that *E. coli* 6S RNA is of similar size and also accumulates during stationary phase [6], we hypothesized that the ∼200 nt band was 6S RNA of *Coxiella*. To address the hypothesis, we first mapped the *ssrS* gene by in silico analysis and unpublished 6S RNA sequence data (kindly provided by Ronald Breaker [31]). The *ssrS* gene is located in the 5′ untranslated region (UTR) of *C. burnetii’s ygfA* locus (encoding formyl tetrahydrofolate cyclo-ligase; CBU_0066) (Fig. 5), a linkage that is highly conserved among γ-proteobacteria [31].

**Figure 6:**
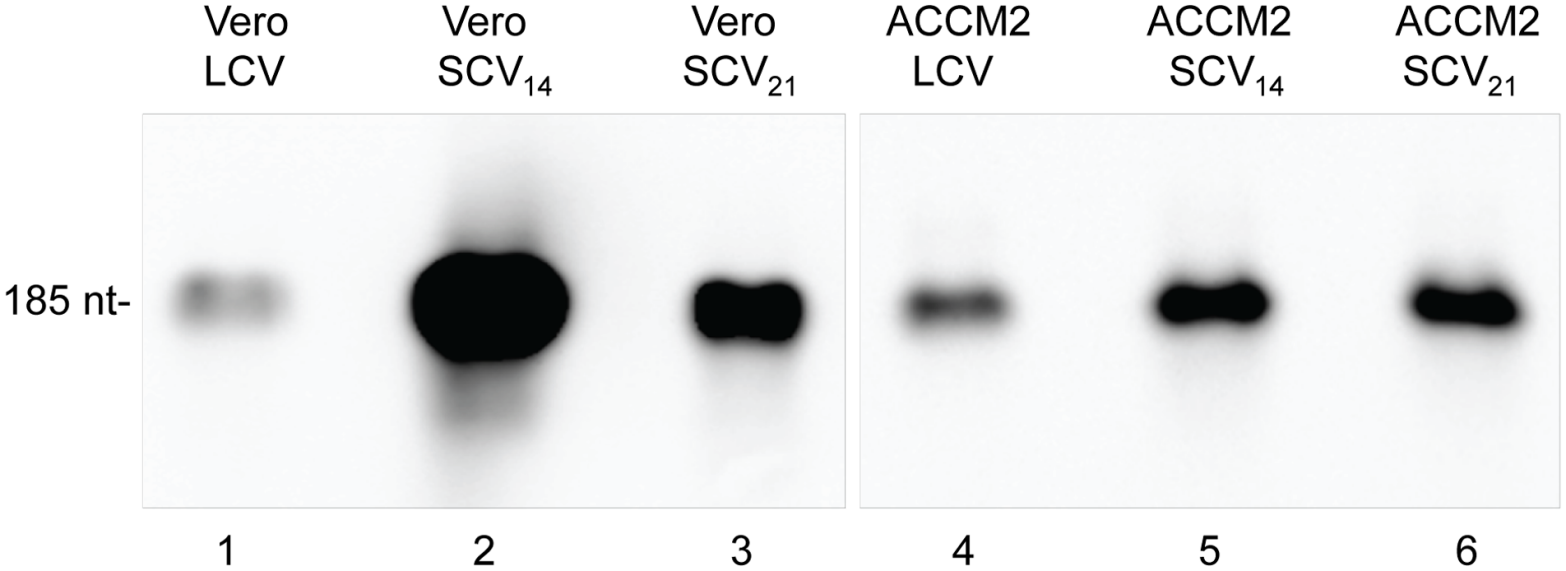
Northern blots showing 6S RNA levels of *C. burnetii*. RNA was isolated from LCVs (3 dpi) and SCVs (SCV_14_, 14 dpi; SCV_21_, 21 dpi) grown in Vero host cells and ACCM2, respectively. Hybridizations were performed at high stringency using a 6S RNA-specific biotinylated oligonucleotide probe. 3 µg RNA was used for all lanes. The size of the signal is indicated to the left.

**Figure 7:**
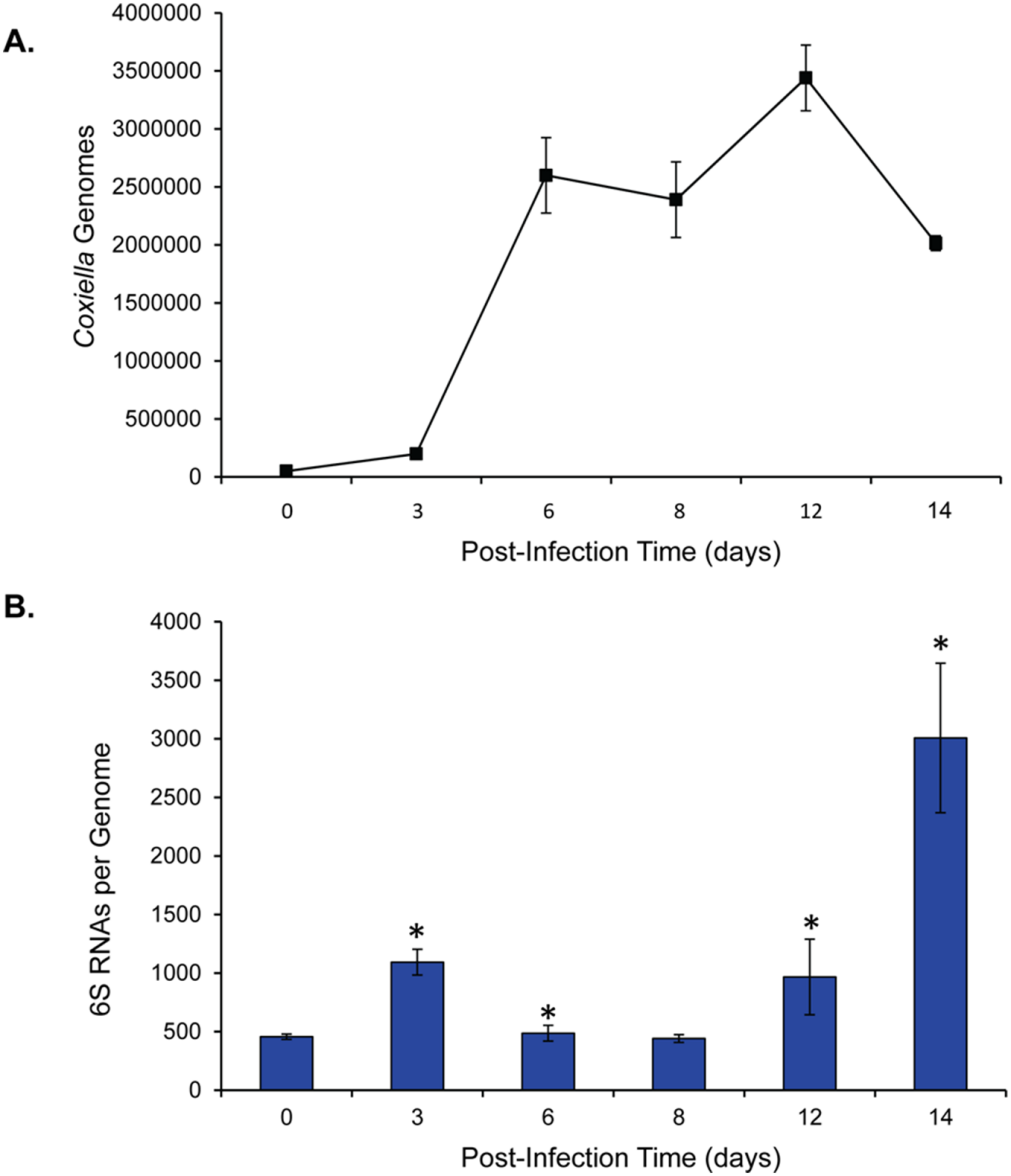
*C. burnetii* 6S RNA copies per genome over a 14-d infection period. **A.** Number of *C. burnetii* genomes over a period of 14 d in infected Vero cells, as determined by qPCR with a primer set specific to *rpoS*. Values on graph represent the means ± standard deviations of the results of 6 independent determinations. **B.** Average number of copies of *C. burnetii* 6S RNAs per genome over a 14-d infection of Vero cells. The number of 6S RNA copies was determined by qRT-PCR using primers specific for 6S RNA and 1 µg total RNA from each time point using the same source cultures as panel A. Values represent the means ± standard deviations of the results of 6 independent determinations. Asterisks denote a significant difference relative to the 0-d sample (p<0.05 by student’s t test).

To confirm the identity of the presumed 6S RNA band, we performed Northern blot analyses of RNA isolated from both morphotypes of *C. burnetii* cultured in Vero cells and in ACCM2. Northern blot analyses were also performed on total RNA isolated from SCVs at 14 dpi and 21 dpi to compare 6S RNA levels at early and late stationary phase. Blots were then probed with a biotinylated RNA designed from the 5′ UTR of *C. burnetii’s ygfA* locus. The resulting Northern blot validated the identity of 6S RNA and the size was observed to be ∼185 nt, which we later confirmed by RNA-seq. Furthermore, 6S RNA was found to accumulate in SCVs relative to LCVs, irrespective of growth conditions (Fig. 6). This is similar to the 11-fold increase reported for *E. coli* 6S RNA at stationary phase versus exponential phase [6]. Levels of 6S RNA in *C. burnetii* cultured in Vero cells were ∼9-fold higher in SCVs at 14 dpi compared to LCVs at 3 dpi when blots were analyzed by densitometry. Levels of 6S RNA dropped ∼2 fold between 14 dpi and 21 dpi (Fig. 6, lane 1 compared to lanes 2 and 3). On the other hand, the transcript level of 6S RNA in *C. burnetii* cultured axenically was ∼2-fold higher in SCVs at 14 dpi compared to LCVs at 3 dpi and then remained stable through 21 dpi (Fig. 6, lane 4 compared to lanes 5 and 6). To further analyze the increased transcript level of 6S RNA during the SCV phase, we used qRT-PCR to quantify and compare *C. burnetii* genome numbers to 6S RNA levels over a 14-d infection period in Vero cells (Fig. 7). Results showed the greatest increase (∼6-fold) in 6S RNA on day 14 as compared to day 0 of the infection period (Fig. 7B). When 6S RNA levels were compared between SCVs isolated from infected Vero cells versus axenic cultures, a ∼7-fold higher transcript level was observed in SCVs isolated from Vero cells (Fig. 6, compared lanes 2 and 5) indicating a potential role for 6S RNA during intracellular growth. A similar observation has been reported for *L. pneumophila*, where 6S RNA was shown to be important for optimal expression of genes during intracellular growth [5].

### 6S RNA Co-immunoprecipitates with RNAP

**Figure 8:**
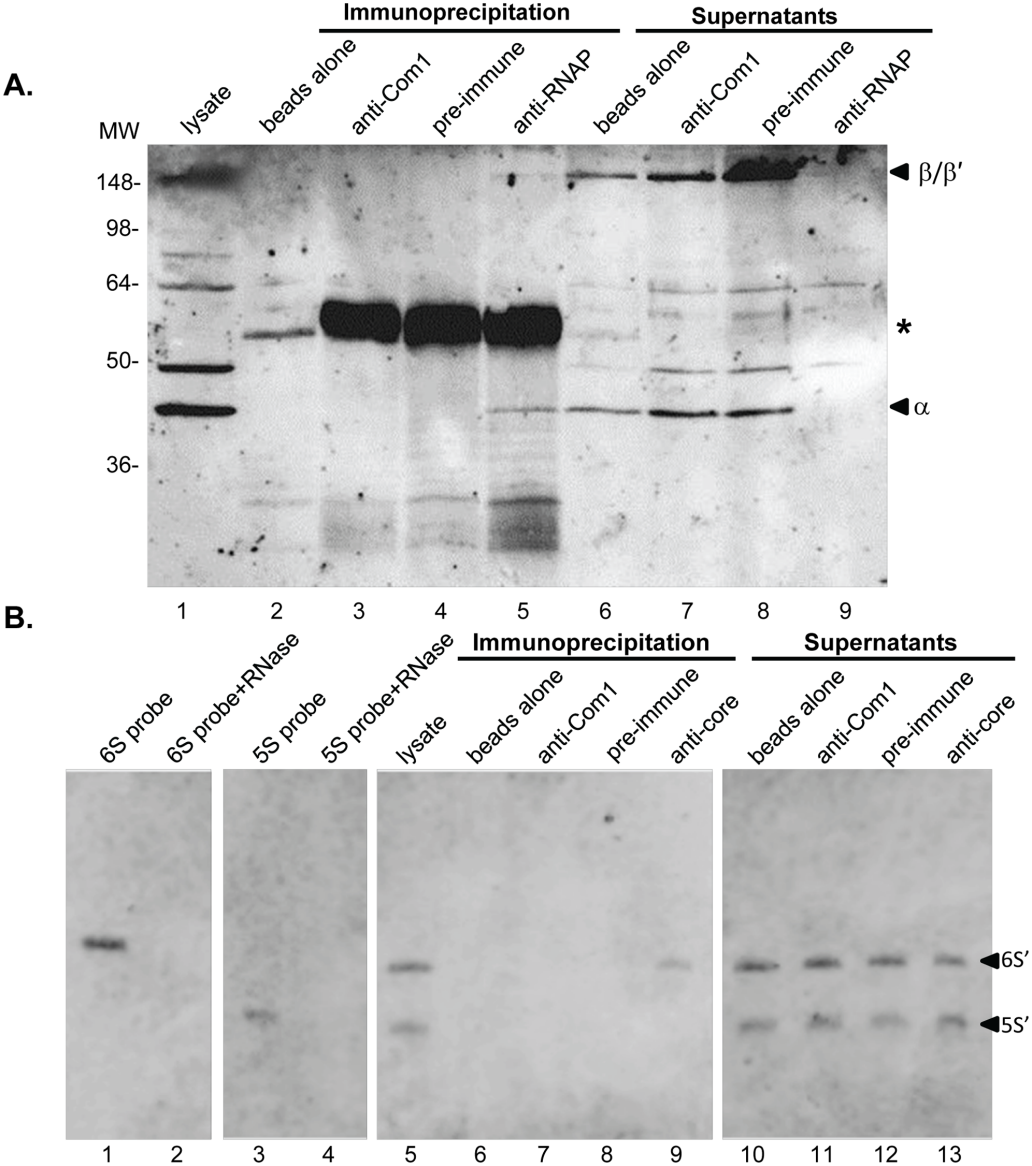
6S RNA co-immunoprecipitates with *C. burnetii* RNAP. **A.** Immunoblot showing IP reactions of a *C. burnetii* lysate and corresponding supernatant samples using various antibodies. IPs were performed with no antibody (lanes 2 and 6), rabbit anti-*Coxiella* Com1 antibody (lanes 3 and 7), pre-immune rabbit serum from the rabbit used to generate anti-RNAP antibodies (lanes 4 and 8) and rabbit anti-RNAP antibody (lanes 5 and 9). The presumed β/β’ and α subunits of RNAP are indicated. Molecular weight values from standards are given to the left in kDa. An asterisk indicates the IgG heavy chain band. **B.** RPAs performed on IP samples. Specific biotinylated probes were used to detect samples containing 6S RNA and 5S RNA. 43 ng of RNA and 4.3 pg probe were used in each RPA reaction, except IP-anti-Com1, where 22.8 ng RNA was used. Lanes 1 and 3 contain untreated 6S RNA and 5S RNA probes, respectively, while Lanes 2 and 4 contain 6S RNA and 5S RNA probes plus RNase, respectively. The RNase-protected portion of the 6S and 5S RNAs (6S’ and 5S’; respectively) are arrowed to indicate the presence or absence of corresponding signals in lanes 5–13.

Previous studies with *E. coli* [6], *Bacillus subtilis* [32] and *L. pneumophila* [5] have shown a physical interaction between 6S RNA and RNAP. To investigate whether this interaction exists in *C. burnetii*, we carried out IP studies using a *C. burnetii* lysate and antibodies that recognize *E. coli’s* core RNAP subunits (a generous gift from Dr. Karen Wassarman, University of Wisconsin-Madison). When IP products were analyzed on western blots, two bands (∼154 kDa and ∼43 kDa) were observed that likely correspond to β/β’ and α subunits of *C. burnetii’s* RNAP, respectively (Fig. 8A, lane 5), based on previous observations in *E. coli* IPs using the same antibody [6]. These two bands were not observed in IPs carried out without antibody, irrelevant antibody (anti-*Coxiella* Com1) or the corresponding pre-immune rabbit serum (Fig. 8A, lanes 2–4, respectively) indicating that the antibody specifically recognizes *C. burnetii’s* RNAP. RPAs on RNA prepared from IP samples showed that 6S RNA was present in IPs prepared using anti-RNAP antibody (Fig. 8B, lane 9) and was absent in controls, indicating that 6S RNA co-immunoprecipitates with core RNAP. Further, a 5S RNA control was detected in IP supernatants but was absent in IP samples, indicating that binding of 6S RNA to RNAP is specific.

## Discussion

Although *C. burnetii* is an obligate intracellular parasite in nature, its life cycle includes a spore-like, dormant SCV morphotype that enables the bacterium to persist and survive outside of host cells. Given the disparate physical conditions encountered by *Coxiella* in the context of the environment and host, it is highly likely that the bacterium employs a rapid and efficient means of regulation to withstand the changing, harsh conditions. Recently, sRNAs have become increasingly recognized as modulators of gene expression, and their role in controlling stress response and virulence, directly or indirectly, has been shown in several bacteria [4], [33]. Here, we describe a deep sequencing-based identification of sRNAs in *C. burnetii*. RNA-seq has been used previously on several other organisms to identify novel non-coding RNAs [34]–[36], but this is the first experimental evidence for, and identification of, sRNAs in *C. burnetii*.

Analysis of sRNA libraries generated from total RNA isolated from *C. burnetii* grown in Vero cells and in axenic medium led to the identification of fifteen novel sRNAs, referred to as CbSRs 1–15. To ensure that the identified sRNAs were authentic, we experimentally verified their existence using Northern blot analyses and identified their strand of origin. However, CbSR 7, although detected by RNAseq, was not detectable by Northern blot analysis [28]. The lengths of most of the CbSRs estimated from Northern blots were in fairly good agreement with that determined by RNA-seq. Moreover, the CbSRs are unique to *C. burnetii,* and with the exception of CbSR 8, highly conserved among six strains of the bacterium. All CbSRs were independently detected in both morphotypes of *Coxiella* isolated from both Vero cells and ACCM2, but their levels changed as a function of growth conditions. These results strongly suggest that CbSRs play a regulatory role in the physiology of *Coxiella.* Not surprisingly, transcript levels of most CbSRs increased during growth phase (LCV) as compared to stationary phase (SCV). A similar observation has been reported in *S. pyogenes*, where transcript levels of most sRNAs are abundant at exponential and early stationary phase as compared to late stationary phase [12]. Based on these observations one could predict that CbSRs help regulate genes that are involved in metabolic functions.

When we compared the transcript levels of CbSRs obtained from *Coxiella* grown in host cells versus axenic medium, eight sRNAs were found to be at higher levels during intracellular growth. Of these, CbSR 12 is particularly striking with regards to its up-regulation in the host cell, and is a current focus of research in our lab. The role of sRNAs in controlling pathogenesis and virulence has been reported in a number of bacteria, including *L. monocytogenes* [37], *Salmonella typhimurium* [38], and *Vibrio cholerae* [39]. We hypothesize that these eight CbSRs are involved in regulating the bacterium’s stress response in the intracellular niche.

Interestingly, using in silico analysis we also discovered that *C. burnetii* lacks an apparent Hfq; an RNA chaperone that modulates translation of many mRNAs and also stabilizes interactions of sRNAs with target RNAs. Previous reports have shown that *hfq* null mutants of pathogens that normally possess Hfq show decreased growth rates, increased sensitivity to stress conditions and impaired virulence [40], [41]. The significance of this observation in *C. burnetii* is unclear. Either *Coxiella’s* sRNAs are Hfq-independent, similar to many Gram-positive bacteria [42], or the bacterium possess an atypical Hfq, as reported for *Borrelia burgdorferi* [43].

In addition to identification of 15 novel sRNAs, we also identified the bacterium’s RNase P RNA (encoded by *rnpB*), tmRNA (encoded by *ssrA*) and 6S RNA by RNA-seq. RNase P RNA and tmRNA are well-studied sRNAs that are conserved among all bacteria. Studies have shown that RNase P RNA is the ribozyme component of RNase P that is involved in processing of 4.5S RNA and tRNA precursor molecules [44]. On the other hand, the tmRNA rescues stalled ribosomes during translation and tags incompletely translated proteins for degradation [45]. 6S RNA is widely distributed among several bacteria, and its biology has been under investigation since its identification in 1976 [46]. Studies in *E. coli* [6], *B. subtilis* [32] and *L. pneumophila* [5] have shown that 6S RNA specifically associates with RNAP and interferes with transcription. Moreover, some functions of 6S RNA include upregulation of genes involved in stress response and nutrient acquisition [5], long-term survival during stationary phase [47] and regulation of *relA* and ppGpp synthesis during stringent response [48]. Considering *C. burnetii’s* intracellular niche, these functions would be clearly beneficial. This encouraged us to further investigate the biology of 6S RNA in *C. burnetii*.

**Figure 9:**
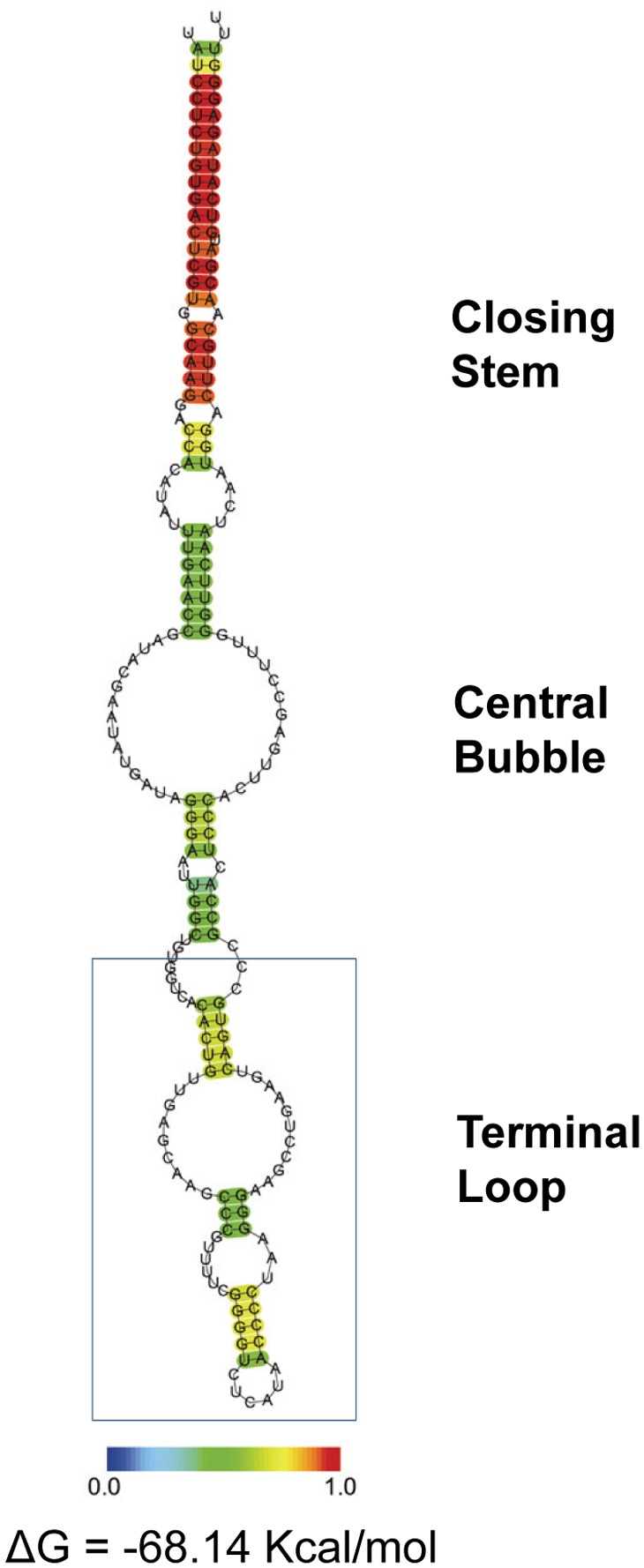
Predicted secondary structure of *C. burnetii’s* 6S RNA as determined by Centroidfold [56]. The color scale at the bottom represents a heat color gradation from blue to red, corresponding to base-pairing probability from 0 to 1. The free energy of the structure is also shown.

The *ssrS* gene of *C. burnetii* was mapped to the 5′ UTR of *ygfA* (Fig. 5). A linkage of *ssrS* and *ygfA* is conserved among many bacterial species [31]. Also, the predicted secondary structure of the *C. burnetii* 6S RNA was found to be highly similar to the published consensus structure of 6S RNA, consisting of a single-stranded central bubble, including two conserved G-C base pairs surrounding the bubble on both sides, flanked by a closing stem and terminal loop (Fig. 9) [31]. The central bubble mimics the structure of a DNA template in an open promoter complex and also occupies the active site of the RNAP. These observations suggest that the 6S RNA of *C. burnetii* is functional.

When we examined the transcript levels of 6S RNA in *C. burnetii* using Northern blot analysis, we found that it was present at much higher levels in the SCV stage of the bacterium, irrespective of growth conditions (Fig. 6). These results were also confirmed by qRT-PCR (Fig. 7). This increase is similar to what has been observed in other bacteria, where 6S RNA reaches its highest abundance during stationary phase [5], [6]. However, a ∼7-fold higher transcript level was observed during intracellular versus axenic growth (Fig. 6, compare lanes 2 and 5). A similar observation has been reported for *L. pneumophila*, a bacterium that is closely related to *C. burnetii*. In fact, deletion of the *ssrS* gene of *L. pneumophila* reduced intracellular growth in host cells by 10-fold, while there was no effect on the mutant’s growth in axenic medium [5]. A recent study in another pathogenic bacterium, *Y. pestis*, also showed increased transcript levels of 6S RNA *in vivo* [36]. Also, the transcript level of 6S RNA in *C. burnetii* grown in Vero cells increased ∼9-fold by 14 dpi (SCV), compared to 3 dpi (LCV), and then dropped ∼2 fold at 21 dpi (SCV). However, this drop was not observed in *C. burnetii* grown axenically. It is possible that, in Vero co-cultures, some SCVs are still intracellular at day 14 (i.e. some Vero cells are extant), whereas by day 21 all the host cells are dead, SCVs are extracellular and 6S RNA falls to a background level. Taken as a whole, our observations are suggestive of 6S RNA’s involvement in regulating genes related to *C. burnetii’s* stress response, especially during intracellular growth. In order to specifically identify genes whose transcript level is altered by 6S RNA, we are currently examining an *ssrS* mutant and a 6S RNA-overexpression strain of *C. burnetii*. Analysis of the transcriptomes of these strains will yield important clues regarding the 6S RNA regulon.

Our studies have also shown that, similar to other bacteria, *C. burnetii’s* 6S RNA associates specifically with RNAP. This was demonstrated by IP experiments using a *C. burnetii* lysate and an antibody that recognizes core RNAP. Western blotting was performed to confirm that the antibodies were specific to *C. burnetii’s* RNAP (Fig. 8A). An RPA was also performed on RNA isolated from the IP samples using 6S RNA- and 5S RNA-specific biotinylated probes. Results clearly showed that 6S RNA was present exclusively in IP samples where RNAP was present (Fig. 8B). This confirms a physical association between 6S RNA and RNAP. Based on these observations and previous research on other bacteria, we predict that 6S RNA alters transcription in *C. burnetii* by associating with its RNAP. The 6S RNA of *E. coli* is known to bind to all forms of RNAP, however, it preferentially interacts with RNAP-σ^70^ [49]. Early work with *E. coli* demonstrated that 6S RNA binding to RNAP-σ^70^ during stationary phase inhibits polymerase binding to certain σ^70^-dependent promoters, thus selectively regulating transcription (reviewed in [50]). Later, it was revealed that the 6S RNA of *E. coli* also activates certain σ^s^-dependent promoters (reviewed in [50]). In contrast, the 6S RNA of *L. pneumophila* was found to serve mainly as a positive regulator of genes involved in amino acid metabolism, stress adaptation, DNA repair/replication and detoxification [5]. Based upon these observations, we predict that *C. burnetii’s* 6S RNA acts as both a positive and negative regulator as cells approach stationary phase (SCV stage).

**Figure 10:**
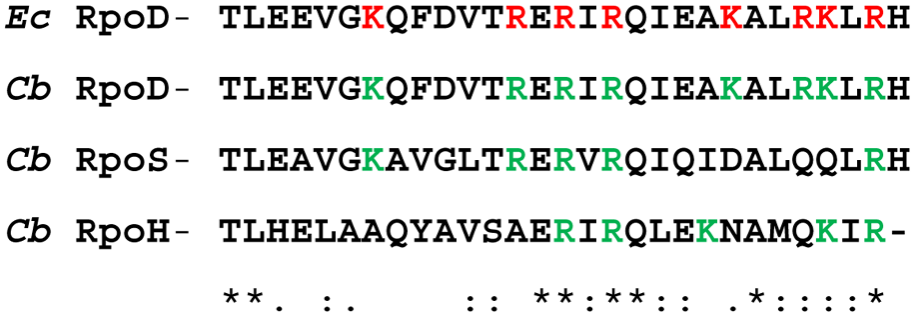
The 4.2 region of *E*. *coli* RpoD and comparison to predicted, homologous regions of *C. burnetii* sigma factors. *E. coli* (*Ec*) and *C. burnetii* (*Cb*) 4.2 regions of sigma factors RpoD, RpoS and RpoH are shown. Positively-charged amino acids of the *E. coli* sigma factor RpoD 4.2 region involved in binding 6S RNA [52] are shown in red. Positively-charged residues in the predicted 4.2 region of *C. burnetii* sigma factors are shown in green. ClustalW alignment results are shown on the bottom line, where an asterisk indicates perfect identity, a colon indicates similar amino acids with conservation and a period indicates weakly similar amino acids with conservation.

RpoS (σ^s^) is classically the major starvation/stationary phase sigma factor, but it serves as the dominant sigma factor during exponential growth of *C. burnetii* [51]. The choice of σ^s^ is thought to be due to the stressful conditions encountered by *Coxiella* in the PV. With this in mind, we were curious about the potential targets of *Coxiella’s* 6S RNA. Eight positively-charged amino acids have been shown to create a surface that is required for binding of 6S RNA to the 4.2 region of *E. coli’s* RpoD (σ^70^) [52]. Analysis of *Coxiella’s* RpoS and RpoH 4.2 regions indicates that they each possess only five positively-charged amino acid residues (Figure 10). This suggests that *Coxiella’s* 6S RNA interactions with RNAP-RpoS and RNAP-RpoH would be minimal or absent. In contrast, the 4.2 region of *Coxiella’s* RpoD (σ^70^) shares 100% identity with 30 amino acid residues of the *E. coli* σ^70^ 4.2 region with all eight positively-charged amino acids present (Figure 10). Taken together, these analyses suggest that *Coxiella’s* 6S RNA would mainly interact with RNAP-σ^70^. Nevertheless, the dominant role of RpoS in the log-phase growth of *C. burnetii* suggests the potential for an atypical mechanism of 6S RNA-mediated gene regulation that warrants additional research.

In the past few years, sRNAs have been identified in many bacteria; however, there are few reports on characterization of their target(s). Various computational and experimental approaches have been employed in order to identify these targets [53]. With the aim of predicting potential roles for the identified CbSRs, we used TargetRNA2 software [54] to predict mRNA targets that could base pair with the sRNAs. However, none showed significant binding to a specific and prominent mRNA target. We can speculate on the roles of some of the sRNAs based on the location of their coding sequence relative to neighboring genes. In the case of antisense sRNAs, the RNA that they regulate could be the corresponding mRNAs. Further, most of the antisense and ORF-derived sRNAs are less abundant than intergenic sRNAs indicating that they preferably base-pair with mRNAs encoded nearby. Unfortunately, since most of these genes are pseudogenes or encode hypothetical proteins, their regulatory function is difficult to predict based on location alone. Interestingly, CbSR 14 is transcribed antisense to the 5′ UTR of *trmE*, a bacterial tRNA modification GTPase that has been implicated in ribosome assembly and other cellular processes including stress response, sporulation and pathogenesis [55]. Since these functions would likely be advantageous to *C. burnetii*, CbSR 14 possibly regulates *trmE*, however, this hypothesis must be experimentally validated. Another probable method of target identification is monitoring the phenotypic changes resulting from experimental manipulation of sRNA transcript levels, and these types of experiments are currently underway.

In conclusion, this study is the first step towards elucidating sRNA-mediated regulation of *C. burnetii’s* physiology and pathogenesis. Further investigations are required to determine the exact role played by each CbSR to help *C. burnetii* transition between the two different cell morphotypes and adapt to the intracellular niche.

## Supporting Information

Table S1 **Sequencing statistics.**
(DOCX)

Table S2 **PCR primers used to make probes.**
(DOCX)

Table S3 **qPCR and qRT-PCR primers.**
(DOCX)

Table S4 **Probes used in Northern blots and RPAs.**
(DOCX)
