# Supplementary material for: Identification of Novel Small RNAs and Characterization of the 6S RNA of Coxiella burnetii
Source: PLoS One. 2014 Jun 20;9(6):e100147. doi: 10.1371/journal.pone.0100147 (PMC4064990; doi:10.1371/journal.pone.0100147)
Supplement: Table S1 — Sequencing statistics. (DOCX) [file pone.0100147.s001.docx]

**Table S1.** Sequencing statistics.

| **Sample** | **Reads mapped** | **Total reads** | **% mapped** |
| --- | --- | --- | --- |
| ACCM2-LCV1 | 31,890,682 | 32,945,220 | 97 |
| ACCM2-LCV2 | 34,064,618 | 35,157,535 | 97 |
| ACCM2-SCV1 | 22,937,399 | 23,682,545 | 97 |
| ACCM2-SCV3 | 29,168,247 | 30,027,641 | 97 |
| VERO-LCV2 | 61,191,633 | 81,206,707 | 75 |
| VERO-LCV3 | 45,315,665 | 65,102,022 | 70 |
| VERO-SCV1 | 33,270,940 | 47,656,046 | 70 |
| VERO-SCV2 | 48,317,348 | 59,069,599 | 82 |
