# Supplementary material for: Identification of Novel Small RNAs and Characterization of the 6S RNA of Coxiella burnetii
Source: PLoS One. 2014 Jun 20;9(6):e100147. doi: 10.1371/journal.pone.0100147 (PMC4064990; doi:10.1371/journal.pone.0100147)
Supplement: Table S2 — PCR primers used to make probes. (DOCX) [file pone.0100147.s002.docx]

**Table S2.** PCR primers used to make probes*.

| **Primers** | **Sequence 5' to 3'** | **Source** |
| --- | --- | --- |
| Cbsr 1_Forward | CTTTCTGAAGAGGTAATCACGAAG | This study |
| Cbsr 1_Reverse | TAATACGACTCACTATAGGGTCCCTACCAAGCAGTTCTGTC | This study |
| Cbsr 2_ Forward | GATGCTGTTCTTCGTAGGC | This study |
| Cbsr 2_Reverse | TAATACGACTCACTATAGGGCGGCTATCGCTTCTTTGC | This study |
| Cbsr 3_ Forward | AAACAAACCTTGATAGAAAGCG | This study |
| Cbsr 3_Reverse | TAATACGACTCACTATAGGGACTACTTGATATTCCCTCTTTACC | This study |
| Cbsr 4_ Forward | GATAGCGTGGTGGGAATCGGTTAC | This study |
| Cbsr 4_Reverse | TAATACGACTCACTATAGGGGGGTTTGGTGCGGTCAAGTGG | This study |
| Cbsr 5_ Forward | CGAAATGAAGAAAAGCAACTC | This study |
| Cbsr 5_Reverse | TAATACGACTCACTATAGGGGCTTGGACTTCCTCTAAATG | This study |
| Cbsr 6_ Forward | GTAACTACGGGCATTCCATCG | This study |
| Cbsr 6_Reverse | TAATACGACTCACTATAGGGCGGTACTAACGGTTTCTCAAGC | This study |
| Cbsr 7_ Forward | CTTACTAGGGGATTTTTTTTACTCG | This study |
| Cbsr 7_Reverse | TAATACGACTCACTATAGGGTCGTTAGTTGAAACATTGAACG | This study |
| Cbsr 8_ Forward | CGAGGTGCTTTAGCCATTG | This study |
| Cbsr 8_Reverse | TAATACGACTCACTATAGGGAGATTGTAACGAGACGATGAAG | This study |
| Cbsr 9_ Forward | GAGTACCGTTATAAACATGGATAC | This study |
| Cbsr 9_Reverse | TAATACGACTCACTATAGGGTGAAGATGGGTGGAAAGCC | This study |
| Cbsr 10_ Forward | TCTTTTAATGAAGCGGGAATGG | This study |
| Cbsr 10_Reverse | TAATACGACTCACTATAGGGGCAACATTGGCACGATGG | This study |
| Cbsr 11_ Forward | GACATAACTAGACATCAGGTG | This study |
| Cbsr 11_Reverse | TAATACGACTCACTATAGGGGATTGGCTGCTGTAATGG | This study |
| Cbsr 12_ Forward | TAGCTGAGGTCTCTAGGATCTTG | This study |
| Cbsr 12_Reverse | TAATACGACTCACTATAGGGGACCTTAGACTACCTCATTACGTTTAG | This study |
| Cbsr 13_ Forward | GTCGTTCCCGTGCGTAGG | This study |
| Cbsr 13_Reverse | TAATACGACTCACTATAGGGGCCGTCTGCTGTAGTATTGAAG | This study |
| Cbsr 14_ Forward | GCTTTGGGAGATGACCTTCGC | This study |
| Cbsr 14_Reverse | TAATACGACTCACTATAGGGGACCGTGAGGACAGCAGTTTG | This study |
| Cbsr 15_ Forward | GAATCGGGAGAAACACCAC | This study |
| Cbsr 15_Reverse | TAATACGACTCACTATAGGGTGCCTTTAGGAGGTCAGTC | This study |
| 6SRNA_Forward | AATATAAGTGTATCCTCTGT | This study |
| 6SRNA_Reverse | TAATACGACTCACTATAGGGGTTATGAGACCCCGAAAAC | This study |
| 5SRNA_Forward | CCACCTGATTCCATTCCGAACTCAGAAG | This study |
| 5SRNA_Reverse | TAATACGACTCACTATAGGGACTTTCGCATGGGAGACC | This study |

*The T7 promoter sequence is underlined.
