# Supplementary material for: Identification of Novel Small RNAs and Characterization of the 6S RNA of Coxiella burnetii
Source: PLoS One. 2014 Jun 20;9(6):e100147. doi: 10.1371/journal.pone.0100147 (PMC4064990; doi:10.1371/journal.pone.0100147)
Supplement: Table S3 — qPCR and qRT-PCR primers. (DOCX) [file pone.0100147.s003.docx]

**Table S3.** qPCR and qRT-PCR primers.

| **Primers** | **Sequence 5' to 3'** | **Source or Reference** |
| --- | --- | --- |
| 6SRNA_Forward | AATATAAGTGTATCCTCTGT | This study |
| 6SRNA_Reverse | TTGAACCCAAAGGCTCAAGTG | This study |
| RpoS_Forward | CGCGTTCGTCAAATCCAAATA | (8) |
| RpoS_Reverse | GACGCCTTCCATTTCCAAAA | (8) |
