# Supplementary material for: Identification of Novel Small RNAs and Characterization of the 6S RNA of Coxiella burnetii
Source: PLoS One. 2014 Jun 20;9(6):e100147. doi: 10.1371/journal.pone.0100147 (PMC4064990; doi:10.1371/journal.pone.0100147)
Supplement: Table S4 — Probes used in Northern blots and RPAs. (DOCX) [file pone.0100147.s004.docx]

**Table S4.** Probes used in Northern blots and RPAs.

| **Probes** | **Sequence 5' to 3'** |
| --- | --- |
| Cbsr 1 | CTTTCTGAAGAGGTAATCACGAAGTTAGGAAACTTTATCTCATGGAGAGAGAAAGGTCTAAGGACAGAACTGCTTGGTAGGGA |
| Cbsr 2 | GATGCTGTTCTTCGTAGGCGGCTTGGCCGCTTGCCAACAACAGGATCGCACCCATCAGGATAGACAAAATGGCGCTGTTACAACGCCCCACCCCGATGATCAACAAAAAGATAATATGCAAAGAAGCGATAGCCG |
| Cbsr 3 | AAACAAACCTTGATAGAAAGCGCGCTAGATTCCCGCCTGCGCGCATAGACGTCAACTTAAGGAGCGAGGTAAAGAGGGAATATCAAGTAGT |
| Cbsr 4 | GATAGCGTGGTGGGAATCGGTTACGGAAGATCGTTCCTGCCATTCTGTCGCAAATGTAGCCACTTGACCGCACCAAACCC |
| Cbsr 5 | CGAAATGAAGAAAAGCAACTCTTGGCCTGTCAAATGCTGCAAGCACAGATCGATCCCTTGTTGATTGCGAAACTAACAGGACTTCATTTAGAGGAAGTCCAAGC |
| Cbsr 6 | GTAACTACGGGCATTCCATCGCGGGTAGATTTAATCAGTCCTTTAAAAAAATTTTGTTGCGCTGCTTGAGAAACCGTTAGTACCG |
| Cbsr 7 | CTTACTAGGGGATTTTTTTTACTCGTTTTCAATTCTATTGAACCGTTCAATGTTTCAACTAACGA |
| Cbsr 8 | CGAGGTGCTTTAGCCATTGGGCAACCCGTTTATTGGACTAGTATGGATAGATTAAATCAAAACAAATTGAGCATGCGAACTGAACAAAGAGATAAAAGGCCACGTCAAATTCCTAAGTGAGAAAGAATGTTTTGCAGCTCGTTTCAGGTCTTGAAGAAGCTTCATCGTCTCGTTACAATCT |
| Cbsr 9 | GAGTACCGTTATAAACATGGATACCCACTAGGTTGATGTTAGGCATTTTTAAAAAGAAATTCTTATTCGAAAAAACGATTTCTTCATCCATGCCGAACTGCGAAGGCTTTCCACCCATCTTCA |
| Cbsr 10 | TCTTTTAATGAAGCGGGAATGGTTGCCATTGGAAACGTGGAGATGCCAGTAGATATGATGCTGGGAGGACCAATGACGTCGGTAACAGGCCCATCGTGCCAATGTTGC |
| Cbsr 11 | GACATAACTAGACATCAGGTGTAACCAAACAATCACGGAGGATCGACAAATGAGAAAAGCACTTGCTAGCGTTGTTGTAATTATATTTGCAGGCTTACTTTGCACGAGTTTACTGACAATCTTTACAGGCAACTCATCAGGTAACCATTACAGCAGCCAATC |
| Cbsr 12 | TAGCTGAGGTCTCTAGGATCTTGGTGGACAAGGAAGTCCTCGGTGTACTCTAAACGTAATGAGGTAGTCTAAGGTC |
| Cbsr 13 | GTCGTTCCCGTGCGTAGGCCGTCTATCGATAGACAAAGAATCGAGGGCTTTCACGGCCGAAGTCACGGGAATCCCGGCTAAGAGGGGCTTGAAGAACACTAACGGTGTTTTTCTTAGCTCCTTAATCTGGGTCCCCCCGACTCGGCCGTGAAGGTTTTGTATTCTTCAATACTACAGCAGACGGC |
| Cbsr 14 | GCTTTGGGAGATGACCTTCGCTGTGAAAATGGGGGTACCGTTACAGGAGTTACTCATAAGAATTGCGGGCTAACCTGGGAGACTAATCCTCCCGAATTCAATTTTTGCCCAAAGGGCCAAACAACAAACTGCTGTCCTCACGGTC |
| Cbsr 15 | GAATCGGGAGAAACACCACTCTTAAAATTACTATTGAAACGATTACCCAAGCAATCTTCCTTGAAGAAAGTAGCAAGTAATCCTCACTATATCGAGATGACTGACCTCCTAAAGGCA |
| 6S RNA | AATATAAGTGTATCCTCTGTGACTCGTGGCAAGGACCACATATTTGAACCGATACGAATATGATAGGGAATTGGCTGTGGTCACACTGTTGAGCAAGCCCGTTTTCGGGGTCTCATAACC |
| 5S RNA | CCACCTGATTCCATTCCGAACTCAGAAGTGAAAACGCTTAGCGCCGATGATAGTGTGGGTCTCCCATGCGAAAGT |
